# Genome‐wide association mapping of *Fusarium langsethiae* infection and mycotoxin accumulation in oat (*Avena sativa* L.)

**DOI:** 10.1002/tpg2.20023

**Published:** 2020-06-08

**Authors:** Julio Isidro‐Sánchez, Kane D'Arcy Cusack, Carol Verheecke‐Vaessen, Amal Kahla, Wubishet Bekele, Fiona Doohan, Naresh Magan, Angel Medina

**Affiliations:** ^1^ UCD Agriculture & Food Science College of Health and Agriculture Science University College Dublin Belfield Dublin 4 Ireland; ^2^ UCD School of Biology and Environmental Science and Earth Institute College of Science University College Dublin Belfield Dublin 4 Ireland; ^3^ Applied Mycology Group Environment and AgriFood Theme Cranfield University Cranfield Bedfordshire MK43 0AL UK; ^4^ Ottawa Research and Development Center Agriculture and Agri‐Food Canada 960 Carling Ave. Ottawa Ontario K1A 0C6 Canada; ^5^ Centro de Biotecnología y Genómica de Plantas (CBGP, UPM‐INIA) Universidad Politécnica de Madrid (UPM) ‐ Instituto Nacional de Investigación y Tecnología Agraria y Alimentaria (INIA), Campus de Montegancedo‐UPM, 28223‐Pozuelo de Alarcón (Madrid) Spain

## Abstract

*Fusarium langsethiae* is a symptomless pathogen of oat panicles that produces T‐2 and HT‐2 mycotoxins, two of the most potent trichothecenes produced by *Fusarium* fungi in cereals. In the last few years, the levels of these mycotoxin in oat grain has increased and the European commission have already recommended a maximum level for of 1000 μg kg^−1^ for unprocessed oat for human consumption. The optimal and most sustainable way of combating infection and mycotoxin contamination is by releasing resistant oat varieties. Here the objective was to determine if we could identify any genomic loci associated with either the accumulation of *F. langsethiae* DNA or mycotoxins in the grain. In each of two years, field trials were conducted wherein 190 spring oat varieties were inoculated with a mixture of three isolate of the pathogen. Mycotoxins were quantified using liquid chromatography–tandem mass spectrometry. Varieties were genotyped using 16,863 genotyping by sequencing markers. Genome‐wide association studies associated 5 SNPs in the linkage group Mr06 with T‐2 + HT‐2 mycotoxin accumulation. Markers were highly correlated, and a single QTL was identified. The marker *avgbs_6K_95238.1* mapped within genes showing similarity to lipase, lipase‐like or lipase precursor mRNA sequences and zinc‐finger proteins. These regions have previously been shown to confer a significant increase in resistance to *Fusarium* species.

**Abbreviations**
BLUPs: Best Linear Unbiased Predictors
DON: Deoxynivalenol
EM: expectation maximization
FDR: false discovery rate
FHB: Fusarium Head Blight
GBS: Genotype by sequencing
GBS: genotyping‐by‐sequencing
GWAS: Genome wide association studies
LOD: limit of detection
LOQ: limit of quantification
MVN: multivariate normal
PCA: Principal component analysis
PVC: polyvinyl chloride
QTL: Quantitative Trait Loci
SNP: single nucleotides polymorphisms
TE: Tris‐EDTA
UHPLC‐MS/MS: ultrahigh performance liquid chromatography‐tandem mass spectrometry

## 1 INTRODUCTION

Fusarium head blight (FHB) of small grain cereals is an economically important disease caused by several species in the *Fusarium* genus*. Fusarium graminearum, F. culmorum, F. poae, F. avenaceum, F. langsethiae* and *F*. *sporotrichioides* are generally the most widespread species affecting spring oats (Nielsen et al., 2011; Pasquali et al., 2016). Besides toxin production, FHB has been associated with yielding losses and low germination capacity (Bjørnstad & Skinnes, 2008). *Fusarium* species produce a wide range of trichothecene mycotoxins in cereal grains, including trichothecenes. (deoxynivalenol (DON), nivalenol, T‐2 and HT‐2 toxins), zearalenone and moniliformin, (McCormick, Stanley, Stover, & Alexander, 2011) all of which are a significant hazard in the food chain (Magan & Aldred, 2007).

*F. langsethiae* (Torp & Nirenberg, 2004) is a producer of T‐2 and HT‐2 mycotoxins (Edwards, Barrier‐Guillot, Clasen, Hietaniemi, & Pettersson, 2009, 2012a; Hofgaard et al., 2016; Imathiu, Ray, Back, Hare, & Edwards, 2013b; Schöneberg et al., 2018; Yli‐Mattila et al., 2008), and has higher preference for oats than for other cereal such as wheat or barley (Divon, Razzaghian, Udnes‐Aamot, & Klemsdal, 2012; Edwards et al., 2009, 2012b; Imathiu, Ray, Back, Hare, & Edwards, 2009; Opoku, Back, & Edwards, 2018). Among the *Fusarium* pathogens, *F. langsethiae* is the least aggressive (Torp & Adler, 2004), and have less competitive ability to colonize than *F. graminearum* (Edwards et al., 2009; Imathiu, 2008). It solely infects the grain (Divon et al., 2012), has limited ability to penetrate the plant cell wall (Imathiu et al., 2009) and it is an asymptomatic pathogen, meaning that does not cause visual head blight symptoms (Imathiu, Hare, Ray, Back, & Edwards, 2010). T‐2 and HT‐2 toxin have higher toxicity to mammals than DON (Rocha, Ansari, & Doohan, 2005); and the European Commission have recommended a maximum level of 1000 μg kg^−1^ for unprocessed oat grain intended for human consumption and a tolerable daily intake of 0.02 μg kg^−1^ body weight (The European Commision, 2013).

Environmental conditions (Kaukoranta, Hietaniemi, Rämö, Koivisto, & Parikka, 2019; Opoku, Back, & Edwards, 2013), cropping factors (Schöneberg et al., 2018), as well as monoculture (Opoku et al., 2013) are most important factors influencing the accumulation of T‐2 and HT‐2 mycotoxins. Nevertheless, the optimal and most sustainable way of combating infection and mycotoxin contamination is by releasing resistant oat varieties (Pirgozliev, Edwards, Hare, & Jenkinson, 2003). However, the selection of *F. langsethiae* resistant oat genotypes have been strongly delayed due to the lack of knowledge about *F. langsethiae* infection. The identification of genomic loci that confer resistance to mycotoxin accumulation would expedite breeding programs to improve the genetic gain per unit of time (Lande & Thompson, 1990). High‐density single nucleotide polymorphism (SNP) genotyping of unrelated individuals can be used to identify causal mutations that have an effect on a phenotype through association mapping studies. In oats, genome‐wide association studies (GWAS) have been reported previously for β‐glucans (Asoro et al., 2013; Newell et al., 2012), crown rust and powdery mildew (Esvelt‐Klos et al., 2017; Montilla‐Bascón et al., 2015; Winkler et al., 2016), barley yellow dwarf virus (Foresman et al., 2016; Winkler et al., 2016), frost tolerance (Tumino et al., 2016) and lodging and plant height (Tumino et al., 2017). There are no reports evaluating GWAS for resistance to *F. langsethiae* mycotoxin accumulation in oats. The aim of this study was to conduct GWAS analysis with a diverse oat population in order to determine if we could identify genomic loci that contribute to variation in mycotoxin levels in F. langsethiae‐inoculated oats.

**Core Ideas**

- Locus *avgbs_6K_95238.1* encodes lipase precursors mRNA which is associated with resistance to fungi.
- *Mr06* Linkage group plays an important role on resistance to *F. langsethiae*.
- Fusarium inoculation around anthesis favor *F. langsethiae* grain contamination in field experiments.

## 2 MATERIALS AND METHODS

### 2.1 Plant material

A total of 190 spring oat varieties were included in this study, selected to capture genetic variability across Europe. Seeds were sourced from the European Avena Database, Nordic Genetic Resource Centre and a subset of the Avena Genetic Resources for Quality in Human Consumption (AVEQ) project. The panel represent cultivars developed through breeding programs released between 1824 and 2015 in major oat producing regions of Europe; Finland (23 varieties), France (21), Hungary (24), Ireland (32), Norway (23), Poland (20), Sweden (21) and United Kingdom (26). Field trials were carried out at University College Dublin Lyons Research Farm, Kildare, Ireland in 2017 and 2018. Seeds were treated with 100 ml ANCHOR (50% v/v in distillated water) fungicidal seed dressing to reduce seed and soil borne infection. Each year, the trial consisted of a 500‐m^2^ plot area containing two replications in which the varieties and field checks were sown in a randomized complete block design. Each variety or check was planted as a 1.5 m row with spacing of 50 cm between each. Crop management practices were conducted following the recommendations for the site.

### 2.2 Fungal material

Three *F. langsethiae* isolates from the UK, ‘Fe2390’, ‘Fe2391’ and ‘Fe2392’ (Table 1), were used as a composite inoculum for the field trails. All three isolates were treated identically for inoculum preparation as follows, and subsequently combined in equal parts for the final composite inoculum solution. *Fusarium langsethiae* colonies were cultured on potato dextrose agar (PDA) in the dark for 7 days at 20–21 °C. Two fungal plugs (1 cm diameter from the fresh culture) were added to an oat media broth (20 g porridge oats boiled in 1 L of water for 20 min; autoclaved at 121 °C for 15 min) and incubated in a shaker (150 rpm; 25 °C) for 7 days to allow sporulation to occur. The mixture was filtered through two layers of sterile muslin to remove mycelia and agar pieces. The remaining spore solution was collected in 50 ml falcon tubes, and centrifuged (400 rpm; room temperature) for 20 min. The supernatant was discarded, and the spore pellet washed twice with 40 ml sterile distilled water (SDW) and centrifuged again under the same conditions. The pellet was resuspended in 15 ml Tween 20 (0.02% v/v in sterile distilled water) and vortexed. The spore concentration of each of the three *F. langsethiae* isolates was determined using a haemocytometer. During the period in which artificial inoculations were performed, approximately 30 days each year, fresh spore solutions were prepared twice a week and the concentrated solutions stored at 4 °C. Immediately before each inoculation, solutions of the three isolates were adjusted to a working concentration of 1 × 10^5^ conidia ml^−1^ in Tween 20 (0.02% v/v in Sterile distilled water) and combined in equal volumes to get the final spore suspension for artificial inoculation.

**TABLE 1:** Description of the three *Fusarium langsethiae* isolates used as a composite inoculum in this study. Growth rate and T‐2 and HT‐2 toxin concentration were measured after 10 days at 25 °C, 0.98 of water activity on oat based medium. ND: Not detected

Isolate	Growth rate (mm d^−1^)	T‐2 toxin concentration (μg g^−1^)	HT‐2 toxin concentration (μg g^−1^)
Fe2390	4.5 ± 0.4	19.1 ± 5.5	0.3 ± 0.1
Fe2391	5.7 ± 0.3	18.9 ± 2.0	1.3 ± 0.4
Fe2392	5.5 ± 0.1	15.5 ± 1.0	ND

Trials were artificially inoculated with the composite inoculum three times during the oat growth, recorded using the Zadoks’ growth scale (GS) 55, 60 and 65 stage (Zadoks, Chang, & Konzak, 1974). A variety was considered to have reached a specific growth stage when over 50% of the plants in the row exhibited this stage. Each inoculation consisted of approximately 100 ml of the composite inoculum sprayed evenly across all panicles of the row using a handheld pressure sprayer (SO402, Solo, Newport News, VA, USA). Irrigation was provided before and after inoculation to create humid conditions in the plot area conducive to *F. langsethiae* growth (Medina & Magan, 2010). A high pressure/low volume irrigation system was custom built and suspended 2 m above ground level spanning the length of the trial area. This consisted of six polyvinyl chloride (PVC) pipes evenly spaced across the 500‐m^2^ plot, which were fitted with fine misting jets (120.64‐BO‐MR, Deker Horticultural Suppliers Ltd, Meath, Ireland) at regular intervals along each pipe. Water was sprayed at intervals of 20 min with 10 min break for 2 hours.

### 2.3 Mycotoxin quantification

Ultra‐high‐performance liquid chromatography‐tandem mass spectrometry (UHPLC‐MS/MS) was performed to measure mycotoxin concentrations at Cranfield University, Cranfield, Bedfordshire MK43 0AL, UK. The metabolites assessed were T‐2 and HT‐2 toxins. Grain samples were homogenised and 100 ± 10 mg of oat flour was weighed into 2 ml eppendorf tubes and extracted with 500 μl of acetonitrile/water/formic acid (79:20.9:0.1, v/v/v) for 90 min at 300 rpm and 25 °C on a rotary shaker (miniShaker VWR, Leighton Buzzard, UK). Afterward, the extracts were centrifuged for 10 min at 22,600 *g* (Centrifuge 5417S, Eppendorf, Stevenage, UK). The supernatants (200 μl) were transferred to HPLC vials containing 250 μl microinserts and stored at −20 °C prior to analysis.

Analysis by UHPLC‐MS/MS was performed with an Exion series UHPLC system coupled to 6500+ qTRAP‐MS system, coupled also with IonDrive Turbo Spray (both Sciex Technologies, Warrington, UK). Chromatographic separation was achieved on a reversed‐phase ACE 3‐C18 column (2.1 × 100 mm, 3 μm particle size; Hichrom) equipped with a C_18_ security guard cartridge (4 × 3 mm, Gemini, Agilent, Santa Clara, CA, USA) maintained at 40 °C. The gradient elution was carried out with water: acetic acid (v:v, 99:1, solvent A) and methanol:acetic acid (v/v, 99:1; solvent B), both supplemented with 5 mM ammonium acetate to promote the formation of ammonium adducts. The applied gradient was 15 min long as described: 0 min, 10% B; 0–2.0 min, 10–40% B; 2.0–10.0 min, 100% B; 10.0–11.50 min, 100% B; 11.50–12.0 min, 5% B; 12.00−15.0 min, 5% B. The flow rate of the mobile phase was 0.3 ml min^−1^, and the injection volume was set to 3 μl. Electrospray ionization (ESI)‐MS/MS was performed in an unscheduled Multiple Reaction Monitoring (MRM) mode in positive and negative mode with a dwell time of 10 ms per Q3 analyzed. The source conditions were set as follows: Curtain gas 40%, Collision Gas Medium, IonSpray voltage −4500 V (in negative) and 5500 V (in positive), temperature 400 °C, ion source gas 1; 60 psi and ion source gas 2; 60 psi, the entrance potential for all compounds was fixed at 10 V. The acquisition of two MRM per analyte were analysed to confirm identity of the identified metabolite. Data were acquired with Analyst Data Acquisition version 1.6.3, and quantification of data was performed using MultiQuant version 3.0.3. Data were transformed by ln_10_[Toxin+1].

### 2.4 Genotyping by‐sequencing and bioinformatic analysis

Genomic DNA of the 190 varieties was extracted from 1‐week old seedlings. Four seeds of each variety were germinated on cotton wool in seed trays and maintained at room temperature. Five millilitres of 0.2% potassium nitrate (KNO_3_) solution was added for each variety to promote germination and growth. After a week, the first leaf (≤ 100 mg wet weight) was removed from a single seedling of each accession and genomic DNA was extracted using the DNeasy Plant Mini Kit (Qiagen, Hilden, Germany), according to the manufacturer's protocol. Final DNA was eluted with 100 μl of Tris‐EDTA (TE) buffer and all samples were stored at −80 °C. The DNA concentrations of samples were measured using a Qubit dsDNA HS Assay Kit and Qubit Fluorometer (both Life Technologies, Carlsbad, CA, USA) according to the manufacturer's protocol and diluted to 20 ng μl^−1^ in TE buffer. Two enzyme (Msti‐PStI) genotyping‐by‐sequencing libraries for oat were produced at Laval University's Genomic Analysis Platform in Quebec, Canada, using genotyping‐by‐sequencing (GBS) technology (Bekele, Wight, Chao, Howarth, & Tinker, 2018; Elshire et al., 2011; Huang, Poland, Wight, Jackson, & Tinker, 2014). Detailed information regarding the construction of the GBS libraries is described in Huang et al. (2014). Haplotag, a tag‐level haplotype‐based genotyping‐by‐sequencing software, which was developed for oat and other polyploid genomes that currently lack reference genomes (Tinker et al., 2016). Haplotag software is a population‐based GBS pipeline that uses the results of the first two steps of the TASSEL‐UNEAK pipeline that convolute raw sequence data and identify 64‐bp sequence tags. The production mode of Haplotag genotype calls used the tag‐sequences of the 190 lines from a potential of 353130 potential tag‐level haplotypes previously identified by a discovery call in 4657 world‐wide cultivated oat lines (Bekele et al., 2018; Tinker, Bekele, & Hattori, 2016). High quality markers were kept by removing the markers with > 5% heterozygous or missing calls and with a minor allele frequency < 5% using TASSEL version 5.0. A total of 16,383 informative markers retained after filtering of which 2,880 markers have known genetic positions on the updated oat consensus map (Bekele et al., 2018; Chaffin et al., 2016). Missing markers were imputed using a multivariate normal (MVN) ‐ expectation maximization (EM) algorithm (Poland & Rife, 2012). The draft sequences of the cultivar GS.7 (Babiker et al., 2015; Oliver et al., 2013) was used to identify perfect matches with GBS alleles. Preliminary annotation of the three scaffolds carrying the perfect matches was produced by using maize and rice gene models within Augustus 2.2.5 and Augustus 2.666.0 software using maize and rice trained models. The amino acid sequences of the predicted genes were blasted against the rice reference proteome sequence (htttp://www.uniprot.org/proteomes/UP000059680).

### 2.5 Fungal genotyping quantification

Total DNA was extracted from the milled flour using an adaptation of the method described by Edwards, Imathiu, Ray, Back, and Hare (2012b). *Fusarium langsethiae* DNA within all oat flour samples was amplified and quantified using a QuantStudioTM 7 Flex Real‐Time PCR system (Applied Biosystems, Foster City, CA, USA), following the protocol of Edwards et al. (2012b) with few modifications. In brief, *F. langsethiae* DNA standards (10^0^–10^−4^ ng μl^−1^) were used initially to create a calibration curve and the limit of quantification (LOQ) was taken as the lowest standard detectable by RT‐PCR (10^−3^ ng μl^−1^). Any *F. langsethiae* DNA values measuring below the LOQ were replaced by LOQ/2. The amplification mix consisted of *F. langsethiae* primer pairs FlangA29 F (5′‐CAAGTCGACCACTGTGAGTACCTCT‐3′) and FlangA95 R (5′‐TGTCAAAGCATGTCAGTAAAGATGAC‐3′) (Nicolaisen et al., 2009) (1 μM) and SYBR Green Jumpstart Taq ReadyMix reagent (Sigma‐Aldrich, St. Louis, MO, USA), which was used according to manufacturer's instructions. The volume of DNA sample in the reactions was 5 μl in a total volume of 12.5 μl. In the negative control, 5 μl of PCR‐grade water was used in place of the DNA sample. Negative controls were run in triplicates in each assay performed. The PCR programme had an initial denaturation at 95 °C for 20 s followed by 40 cycles with 1 s at 95 °C and 20 s at 62 °C. Following amplification, the melting curves were acquired by heating samples to 95 °C for 15 seconds, cooling to 60 °C for 1 min and then raising the temperature to 95 °C at a ramp rate of 0.05 °C s^−1^ with continuous measurement of fluorescence.

### 2.6 Statistical analysis

Association analysis were evaluated with SNPs placed on the consensus linkage map of Chaffin et al. (2016). Genome‐wide association analyses analysis were performed in R version 3.5.3 (R Core Team, 2019) using rrBLUP package (Endelman, 2011). Principal component analysis (PCA) was performed to examine the level of genetic structure in the panel via singular value decomposition using method developed by Gauch, Qian, Piepho, Zhou, and Chen (2019). The genetic relatedness among genotypes was evaluated via the additive variance‐covariance genomic relationship matrix (kinship matrix, K). The general equation to perform the GWAS was based on the mixed linear model (MLM) described by Yu et al., 2006:

y=Xβ+Zg+Sτ+ε

where *y* is a vector of phenotypes, *β* is a vector of fixed effects that can model both environmental factors and population structure. The variable *g* models the genetic background of each line as a random effect with $\mathrm{Var}\left[g\right]=K\sigma_{g}^{2}$. The variable *τ* models the additive SNP effect as a fixed effect. The residual variance is $\mathrm{Var}\left[e\right]=I\sigma_{e}^{2}$. *X*, *Z* and *S* are design matrices and *K* and *I* represent the kinship and identity matrix, respectively; $\sigma_{g}^{2}$ and $\sigma_{e}^{2}$ are the variance of the markers and variance of the errors.

Mixed linear models incorporating a kinship matrix alone and the first three principal components (PCs) alone were compared with MLMs incorporating both Kinship and the three PCs and a general linear models without covariates (Naïve model). Quantile–quantile plots were used to compare statistical models for their ability to correct for the probability of incorrectly rejecting the null hypothesis when it is true (Type I error inflation) due to population structure and cryptic relatedness (Supplemental Figure S1). To account for multiple testing we applied both the false discovery rate (FDR) and Bonferroni correction at α = 0.05. The threshold for significance for FDR was 1.1 × 10^−5^ when the number of markers was considered (Benjamini & Hochberg, 1995), and Bonferroni cut‐off was establish dividing alpha to the number of markers. A marker was deemed statistically significant if its *p*‐value was lower than the corresponding FDR or Bonferroni value.

## 3 RESULTS

### 3.1 Phenotypic evaluation

Table 2 shows the summary statistics of the phenotype data. The sum of T‐2 and HT‐2 values ranged from 4.89 to 11458 μg kg^−1^ with an average of 429.6 μg kg^−1^. HT‐2 toxin was the main contributor to increase the sum of the toxin with an average of 315 μg kg^−1^. Fungal biomass ranged from 0 to 176 pg ng^−1^. Best linear unbiased predictions (BLUPs) results from the sum of toxin values (T‐2 + HT‐2) ranged from −1.22 to 1.52 (Table 2). After transformation of the data (ln_10_[Toxin+1]), graphical representation of the residuals and the correlation between the residuals and fitted values were diagnosed to check model assumptions following a published protocol (Isidro‐Sánchez, Akdemir, & Montilla‐Bascón, 2017). There was no evidence of correlation between the residuals and the fitted values and residuals were normally distributed.

**TABLE 2:** Summary of the *Fusarium langsethiae* DNA and T‐2 + HT‐2 toxins levels detected in 2017 and 2018 field trials. BLUPs values are showed in parenthesis

Phenotype	Mean	Median	Minimum	Maximum
*Fusarium langsethiae* DNA (pg ng^−1^)	0.38 (0)	0.03 (0)	0 (−0.6)	176 (0.51)
T‐2 toxin (μg kg^−1^)	113 (0)	4.89 (0)	4.89 (−0.42)	4,885 (0.73)
HT‐2 toxin (μg kg^−1^)	315 (0)	53.4 (0)	0 (−1.35)	9,080 (1.59)
T‐2 + HT‐2 (μg kg^−1^)	430 (0)	78.0 (0)	4.89 (−1.22)	11,458 (1.52)

**FIGURE 1:**
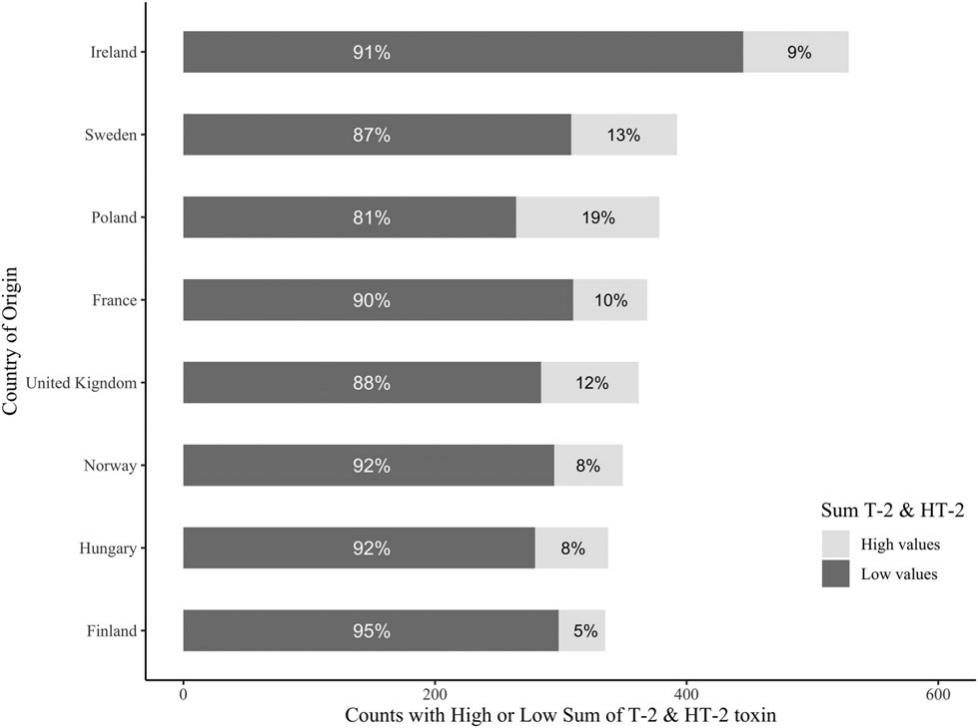
Bar graph showing relative counts and percentage of varieties with high and low mycotoxin load (sum of T‐2 and HT‐2 toxin), relative to country of origin. Grey colours show the number of samples with values below the European recommendation intake of 1000 μg kg^−1^ (Low) or above (High) for unprocessed oat grain intended for human consumption. The percentage of counts in each class is indicated by numbers

The selection of lines across European countries was based on increasing the diversity of the germplasm set (Figure 1). On average, 10.5% of all samples among countries showed toxin values higher than the recommended threshold. Ireland showed the highest number of samples with toxins followed by Sweden. Poland showed the highest samples number above the threshold (19%) followed by Sweden. Finland showed the lowest counts number and the lowest infections above the threshold (5%).

### 3.2 Population structure

**FIGURE 2:**
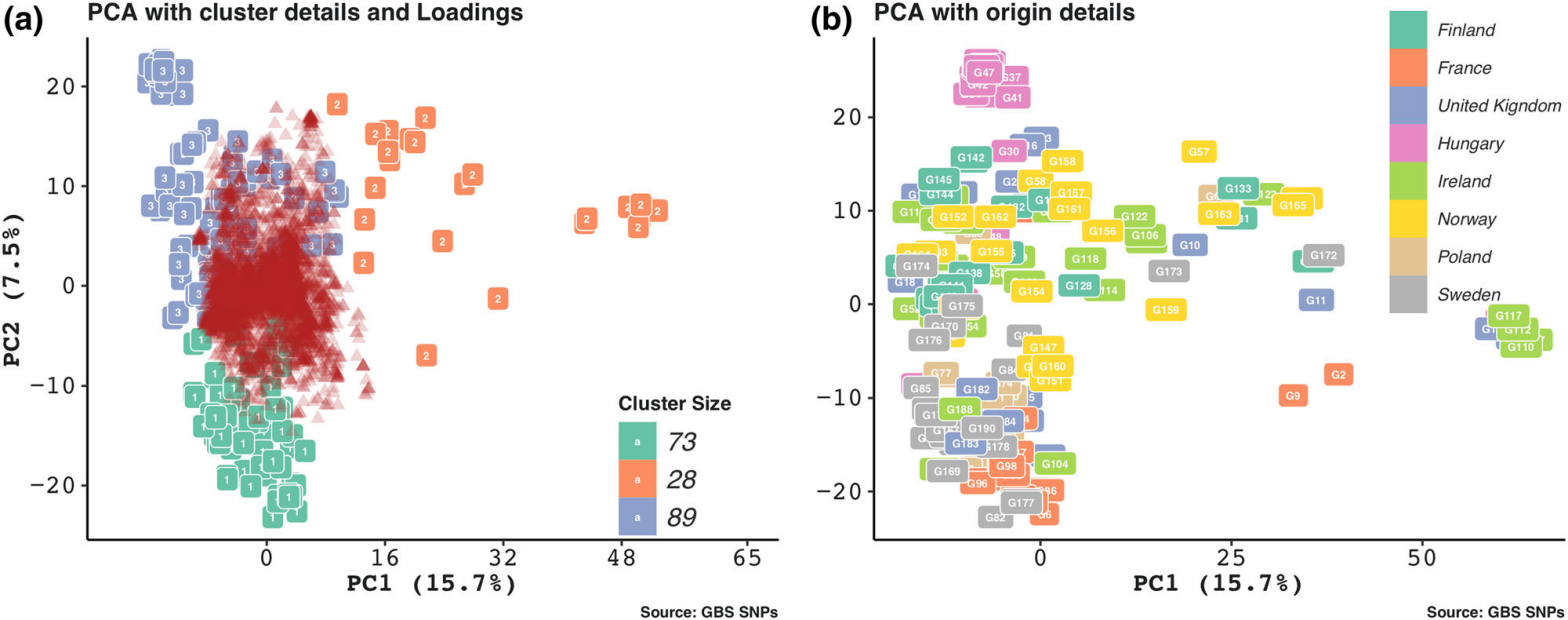
(a) Principal component analysis with the cluster and loadings of markers (red triangles) information. (b) PCA by origin. PC1: Principal component 1. PC2: Principal component 2. The level of population structure was explored to gain insight into its possible effect on the GWAS analysis

Principal component analysis (PCA) was performed following recommendations of Gauch et al. (2019) in a double centered matrix (Figure 2). The PCA showed that the first two axes accounted for 15.7% and 7.5% of the total genetic variation in the dataset. This indicated that the population structure (PC) among the lines was low as compared with other species (Guo et al., 2014). Cluster analysis indicated that there were small groups (Figure 2). The first group contains 73 individuals, mostly from France, Poland and Sweden. Group 2 contains 28 genotypes mainly from England and Norway and group 3 mainly from Hungary, Ireland and Finland. Mean sum T‐2 + HT‐2 values for the groups were 4.6, 4.2, 3.8 μg kg^−1^ for groups 1, 2, and 3 respectively.

### 3.3 Association analysis

**FIGURE 3:**
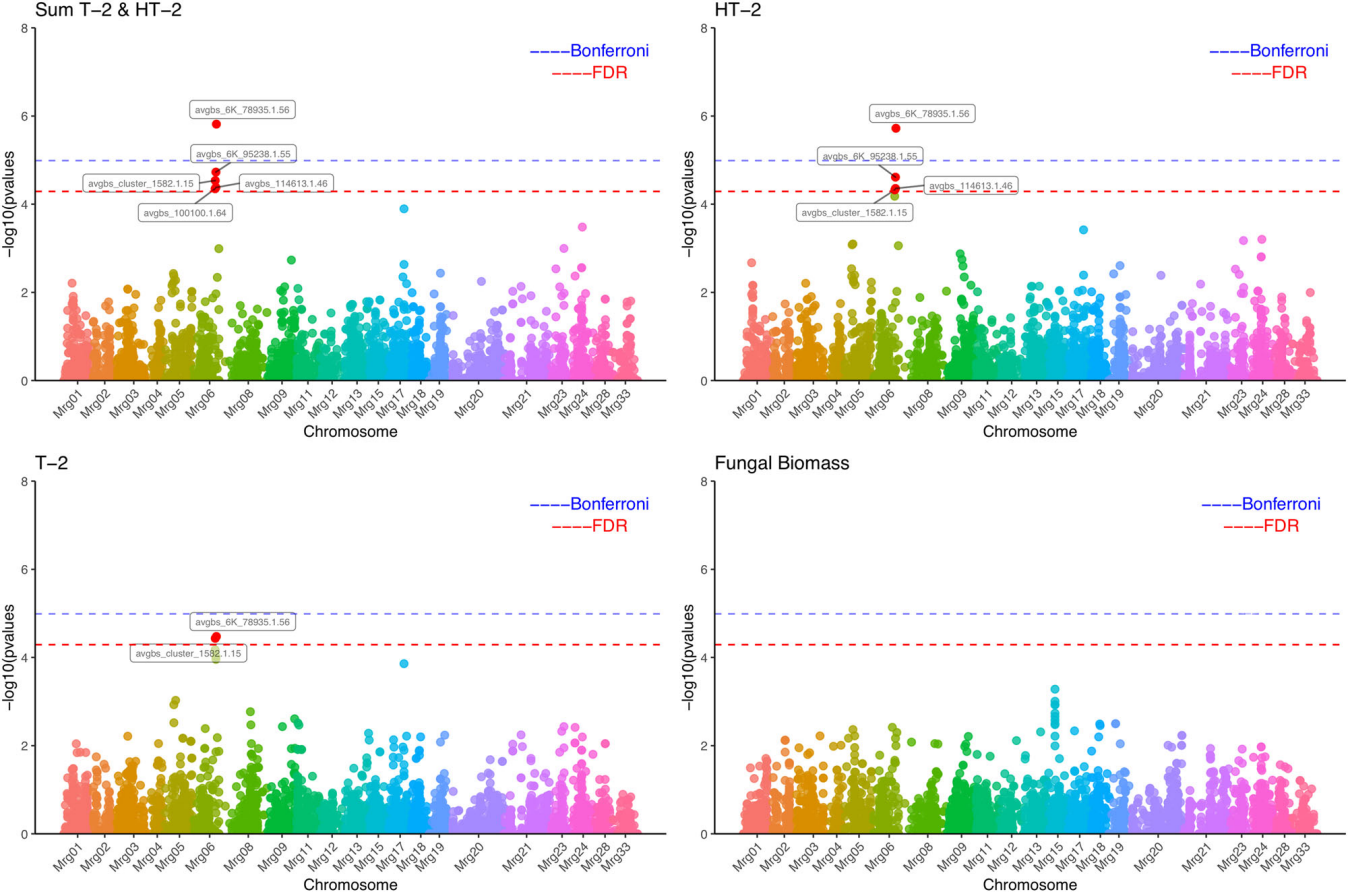
Manhattan plots of ‐log10*p*values for mycotoxins and fungal biomass using kinship and principal components as covariates. Horizontal dotted lines represent the genome‐wide significance threshold under Bonferroni and False discovery Rate (FDR)

As expected from the evaluation of single factors in the mixed model, models that did not include the transformation of the data and Q or K identified a large number of significant, false positive markers (Supplemental Figures S2 and S3). Given such large numbers of significant markers that are likely false positives, these models were excluded from further analyses and the number of significant markers were greatly reduced with the use of transform data and the addition of Q and K in the mixed model. There were 4,988 SNPs with known positions on the consensus linkage map of Chaffin et al. (2016) after data filtering. The MLM incorporating a kinship matrix and the first three PCs as covariates produced an observed‐to‐expected *p*‐value relationship most closely aligned with expectations under the null (Figure 3; Supplemental Figure S1) and therefore this models was used in further analyses. Models that did not include K identified a large number of significant, false positive markers (Supplemental Figures S2, S3 and S3).

Five statistically significant associations between SNP genotypes and *F. langsethiae* mycotoxin levels were detected in linkage group Mrg06 from 99 to 104.5 cM (Table 3). Statistically significant associations between SNP genotypes were detected for all traits except for fungal biomass (Figure 3). Single‐nucleotide polymorphisms, *avgbs_cluster_1582.1.15* and *avgbs_6K_78935.1.56*, were consistently detected for T‐2, HT‐2 and the sum T‐2 + HT‐2 (Figure 3).

**TABLE 3:** List of significantly associated markers for mycotoxin contamination

Locus Name	−log10*p*values	Group	Position
*avgbs_cluster_1582.1.15*	4.53	Mrg06	99.1
*avgbs_6K_78935.1.56*	5.81	Mrg06	104.5
*avgbs_6K_95238.1.55*	4.72	Mrg06	102.3
*avgbs_100100.1.64*	4.35	Mrg06	98.5
*avgbs_114613.1.46*	4.38	Mrg06	102.2

Significant hits were highly correlated from 0.94 to 0.99 Pearson correlation (Supplemental Figure S5). The highest correlation appeared between *avgbs_100100.1.64* and *avgbs_cluster_1582.1.15*.

### 3.4 Identification of physical positions and sequence homology

Four out of the five significantly associated markers had perfect matches on GS scaffolds that are carrying multiple GBS alleles from Mrg06 of the consensus map. All four had only one perfect match except avgbs_6K_95238.1. Alleles of the locus avgbs_6K_95238.1 had three perfect matches on two scaffolds. Four of the GBS tag sequences had perfect matches on the draft assembly. Only avgbs_6K_95238.1 had a perfect GBS match with the *A. atlantical* genome sequence (Maughan et al., 2019). The markers avgbs_100100.1.64 and avgbs_114613.1.46 have perfect matches on the same scaffold, and inside a genomic region predicted to code for an antioxidant gene. This gene is similar to a rice E3 ligase gene that plays a role in abiotic stress tolerance (Fang et al., 2015). The alleles of avgbs_6K_95238.1 were identified in gene coding regions that shows high similarity to lipase like proteins. The summary of the results are shown on Table 4. Lipases play a significant role in wheat and rice disease reactions (Gao et al., 2017; Gottwald, Samans, Lück, & Friedt, 2012). Besides, one of the significant BLASTp hits was the rice gene Os08g0143600 (e‐value = 3 × 10^−11^), and it is described as similar to Avr9/Cf‐9 rapidly elicited protein. These groups of genes play an essential role in plant defence and immunity (Rowland et al., 2005). Another interesting predicted genomic region that harbours avgbs_6K_78935.1.56 alleles has significant protein similarity with a peroxidase coding gene (Os03g0368000) that is relevant for biotic stress response including reactive oxygen species scavenging (Sasaki et al., 2004). The fact that the GWAS hits are in predicted gene coding regions can be explained by the use of PstI that is known to be a methylation sensitive enzyme in plant genomes (Fellers, 2008). This study identified new associations and candidate genes, which calls for further genetic analysis with the aid of completed genome sequences and molecular studies.

**TABLE 4:** Summary of the GBS hits with perfect matches on GS7 scaffold

LG	Position^a^	GBS_ID	Scaffold ID	GBS start^b^	GBS end^b^	Scaffold Size	Predicted^C^
Mrg06	102.3	avgbs_6K_95238.1	Sc80uBt_39063; HRSCAF = 43996	5374	5437		
Mrg06	102.3	avgbs_6K_95238.1	Sc80uBt_39063; HRSCAF = 43996	37992	38055	53799	5
Mrg06	102.3	avgbs_6K_95238.1	Sc80uBt_4416; HRSCAF = 5921	23463	23526	1367770	182
Mrg06	104.5	avgbs_6K_78935.1	Sc80uBt_57598; HRSCAF = 67022	98294	98357		

a in consensus map.

b GBS perfect match start and end.

c Number of predicted genes.

**FIGURE 4:**
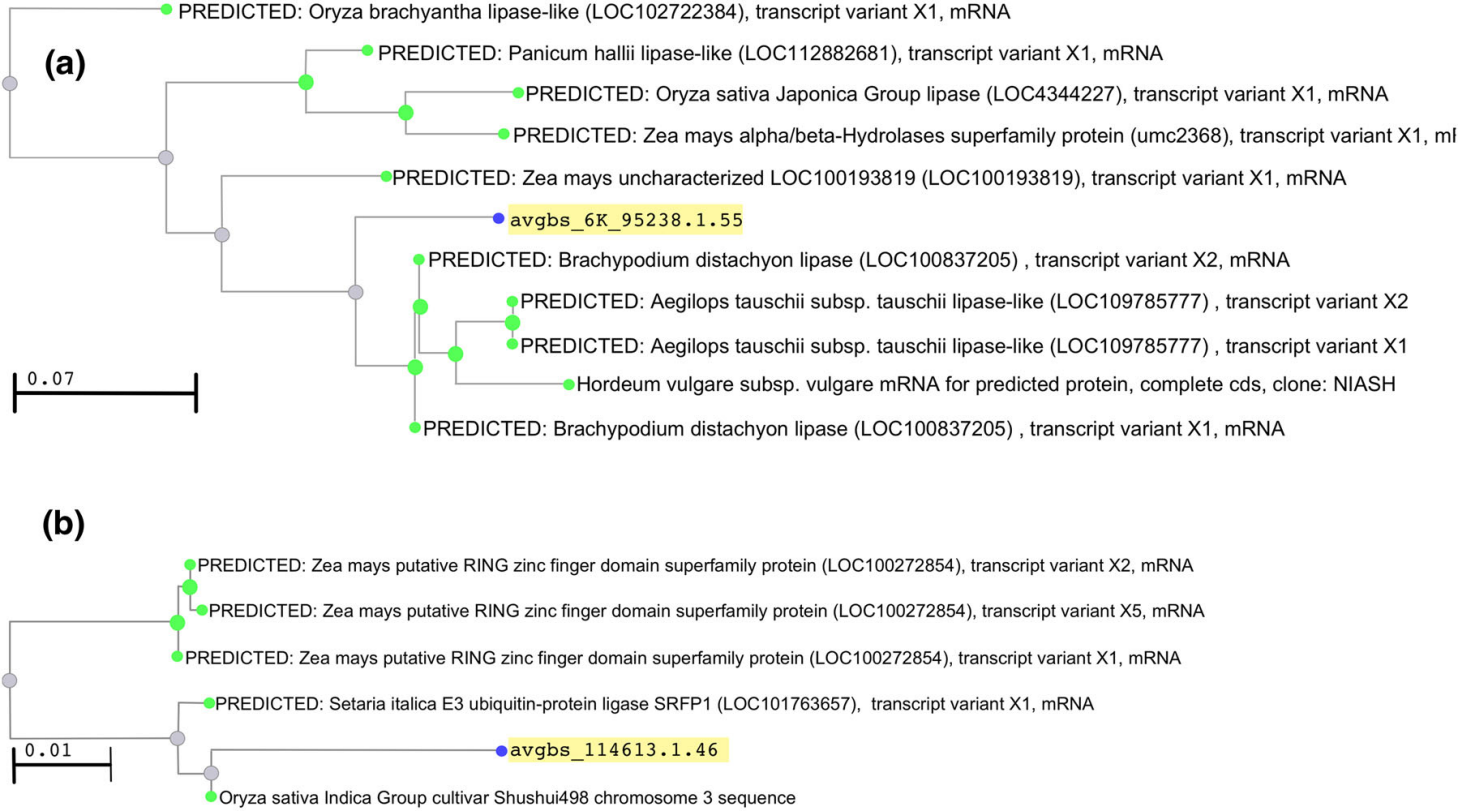
Dendrograms for two significant markers detected in this analysis; (a) *avgbs_6K_95238.1.55* and (b) *avgbs_114613.1.46*. The predicted protein is a lipase (class 3) (Matsumoto et al., 2011). Dendrograms were constructed using NCBI Genome Workbench version 2.13.0 (https://www.ncbi.nlm.nih.gov/tools/gbench/)

Although there were many more alignments to genome sequences of several species, including two hits to *Aegilops tauschii* subsp. *tauschii*, one to *Hordeum vulgare* subsp. *vulgare* and *Phyllostachys edulis*, the majority of these alignments were to lipase, lipase‐like or lipase precursor mRNA sequences (Figure 4). *avgbs_114613.1.46* showed 41 hits, of which *Oryza sativa* (rice) chromosome 3 sequences and *Zea mays* zinc finger were the most significant.

## 4 DISCUSSION

In this study, high rainfall and low temperatures around anthesis favoured the growth of *F.langsethiae* (Supplemental Figure S4) and increased T‐2 and HT‐2 contamination. This is in agreement with recent studies (Hjelkrem et al., 2018; Kaukoranta et al., 2019; Schöneberg et al., 2019) that associated cold temperatures before anthesis and warm temperatures during the 3‐weeks after anthesis with increased grain infection and toxin levels. The 2018 environment was hotter and drier during anthesis, which resulted in lower grain infection (Supplemental Figure S4). As the inoculum level of *F. langsethiae* determines the level of T‐2 + HT‐2 (Kaukoranta et al., 2019), plants were inoculated at three different growth stages. HT‐2 toxin was the main contributor to increase the total toxin (Table 2) probably because in oat T‐2 is quickly converted to HT‐2 as a result of the removal of the acetyl group at C‐4 position of T‐2 (Meng‐Reiterer et al., 2016). Previous field studies at field have shown that fungal biomass of *F. langsethiae* was correlated with T‐2 and HT‐2 concentration (Imathiu, Edwards, Ray, & Back, 2013a; Schöneberg et al., 2019). In this study, different results were observed, and this can be due to the diversity of oat cultivars studied in this study. Depending on the adaptation of the cultivars to specific environmental conditions, the reaction to *F. langsethiae* may be affected differently, which can lead to heterogeneity in T‐2 and HT‐2 patterns in different cultivars. It has already been previously shown that slight modification in *Fusarium* environment can lead to changes in mycotoxin production profile (Medina & Magan, 2011; Shi et al., 2016).

The complexity of the oat genome and the fact that most useful traits in oats are quantitative, controlled by multiple genes, is a limitation in oat genomics, especially because the number of tools developed in oat is few in comparison with other cereals as wheat, maize or rice. From mixed genetic background, the genome association analysis which involves detection of random set of genotypes, genes, and QTL is an effective tool for gene discovery. The keys for success to GWAS are high statistical power, low probability of Type I error, and the study of confounding effects due to population structure and cryptic relatedness that can cause spurious associations (Visscher et al., 2017 and references within). In this study, oat genomic data in combination with mycotoxin phenotypic information was used to examine linkage related marker trait associations. We performed four models accounting for population structure (“Q”), and the genetic similarity (“K”) which play an important role in identifying marker‐phenotype associations. In our analysis, using mixed model equations including both the Q and K offered the best control of type I errors (Figure 3). Principal component analysis showed a moderate level of structure (Figure 2), which taking into account that this collection was consciously formed with diverse oat origin to achieve high genetic diversity, the moderate level of structure was not expected. Population structure in oats is weak (Montilla‐Bascón et al., 2015; Newell et al., 2012; Tumino et al., 2017; Winkler et al., 2016) in comparison with other cereals (Guo et al., 2014; Hamblin et al., 2010) probably due to admixtures and spring and winter types inter‐breeding species (Newell et al., 2012). In the literature, there are numerous GWAS for oats (Asoro et al., 2013; Esvelt‐Klos et al., 2017; Foresman et al., 2016; Montilla‐Bascón et al., 2015; Newell et al., 2012; Tumino et al., 2017, 2016; Winkler et al., 2016) but date there have been no GWAS on *F. langsethiae* resistance in oat. In this study, we evaluated the resistance to *F. langsethiae* in a diverse panel of 190 oat varieties from eight different European locations based on fungal FNA and mycotoxin accumulation in the grain. Five SNPs exhibited strong association with mycotoxin production to inoculation with specific *F. langsethiae* isolates. The high correlation between markers (Figure 4) indicated that there is a strong QTL (Q*fl*.UCD.06) in Mrg06 region (98.5–104.5 cM). The genetic architecture of *F. langsethiae* resistance in oat seems to be complex, since besides this Q*fl*.UCD.06 region on Mr06, there are other regions (Mrg09, 17 and 24) that showed signals but probably due to sample size they were not strong enough to be significant (Figure 4). Two of the five markers were significantly identified by BLASTn against NCBI databases, showed similarity to lipase, lipase‐like or lipase precursor mRNA sequences and zinc‐finger proteins. Lipases are esterases that can hydrolyse long‐chain acyl‐triglycerides into di‐ and monoglycerides, glycerol, and free fatty acids at a water/lipid interface. A typical feature of lipases is “interfacial activation”, the process of becoming active at the lipid/water interface, and this has been related to the plant immune defence reaction. It has been shown that a specific requirement or interaction between fungi and host is needed to have seed infestation, and significant advances have been made to understand the role of several factors controlling mycotoxin biosynthesis (Gao & Kolomiets, 2009; Wong & Schotz, 2002). In this sense, lipid‐derived secondary metabolites produced by host plants are crucial signals that modulate host‐pathogen communication and play an important role to response to biotic and abiotic stresses for acclimation (Shah, 2005; Upchurch, 2008). In wheat, the overexpression of lipids transfer protein genes can indeed improve resistance to *Fusarium* (Zhu et al., 2012), and more recently Schweiger et al., 2016 identified genes on the GDSL lipase family (Akoh, Lee, Liaw, Huang, & Shaw, 2004) that confer a significant increase in resistance to *Fusarium graminearum* and the associated mycotoxin deoxynivalenol (DON). These results indicate a possible role of this lipase‐encoding gene in the significant resistance to *F. graminearum* in wheat. In our study, we found that our significant markers were closely aligned to lipase, lipase‐like or lipase precursor sequences. Markers found in this study showing the strongest association provide an initial step for further studies to find genes associated with resistance to *F. langsethiae*.

## AUTHOR CONTRIBUTIONS

JIS conceived the idea, analysed the data, wrote a large part of the article, prepared the figures. KDC wrote a large part of the article, perform all field phenotypic measurements, and extraction of DNA. CVV perform LC‐MS mycotoxin quantification. WAB did the genotyping GBS calls and bioinformatic analysis. CVV, WAB, AK, FD, NM, AM contributed through discussions of the concepts and ideas and revision of the submitted manuscript.

## CONFLICT OF INTEREST

The authors declare no conflicts of interest.

## Supporting information

Supplemental Material

Supplemental Material

Supplemental Material

Supplemental Material

Supplemental Material
